# Superparamagnetic iron oxide as a tracer for sentinel lymph node detection in uterine cancer: a pilot study

**DOI:** 10.1038/s41598-020-64926-0

**Published:** 2020-05-14

**Authors:** Kosuke Murakami, Yasushi Kotani, Ayako Suzuki, Hisamitsu Takaya, Hidekatsu Nakai, Mitsuru Matsuki, Takao Sato, Masaki Mandai, Noriomi Matsumura

**Affiliations:** 1 Department of Obstetrics and Gynecology, Kindai University Faculty of Medicine, Osaka, Japan; 2 Department of Radiology, Kindai University Faculty of Medicine, Osaka, Japan; 3 Department of Pathology, Kindai University Faculty of Medicine, Osaka, Japan; 4 Department of Gynecology and Obstetrics, Kyoto University Graduate School of Medicine, Kyoto, Japan

**Keywords:** Cancer, Surgical oncology

## Abstract

Sentinel lymph node (SLN) mapping using dye or radioisotopes has been performed in patients with uterine cancer. Superparamagnetic iron oxide (SPIO) can be handled safely and is taken up by lymph nodes (LNs); however, its efficacy in detecting SLNs in uterine cancer remains unknown. This pilot study evaluated the use of SPIO as a tracer for SLN detection in patients with uterine cancer. SPIO was injected into the uterine cervixes of 15 patients with uterine cancer scheduled for pelvic LN dissection. Magnetic resonance imaging (MRI) was performed preoperatively. Five patients also underwent radioisotope injection and single-photon emission computed tomography/computed tomography. Dissected LNs were stained with iron and examined pathologically. Of the radioisotope-positive LNs, 92% were also SPIO/MRI-positive. SPIO/MRI and iron staining were positively correlated. SLNs were identified by iron staining in 93% of cases. Iron staining was strongly positive in two of the five areas of LN metastasis; these were considered SLNs. Staining was negative or very weak in the other three areas and lymph flow disturbance was considered. SPIO and radioisotopes are taken up similarly by SLNs. SPIO/MRI and iron staining may thus be useful for detection of SLNs and diagnosis of LN metastasis in patients with uterine cancer.

## Introduction

Cervical cancer accounts for the third highest number of new cancers and cancer-related deaths among women worldwide, with an estimated 570,000 new cases and 310,000 deaths annually^1^. In addition, the estimated number of new cases of endometrial cancer annually is 380,000^1^. Uterine cancer is therefore an important issue for women worldwide.

Lymph node (LN) metastasis is the most common form of metastasis of both cervical and endometrial cancers and determines the prognosis of patients with these cancers. LN dissection has thus been a standard surgical procedure for uterine cancer^2,3^. However, because LN dissection can cause lymphedema, it is beneficial to identify the first metastatic LN, or sentinel LN (SLN), and remove only this LN. SLN mapping has been applied clinically in many cancers, especially breast cancer^4^. The National Comprehensive Cancer Network guidelines indicate that SLN mapping may be considered for early-stage cervical and endometrial cancers^5,6^. Radioisotopes and dyes, alone or combined, have been used as tracers for SLN mapping^7–10^. A recent prospective study and meta-analysis also reported the use of indocyanine green (ICG)^11,12^. Dyes and ICG have the advantage that the lymph flow can be visualized easily by intraoperative administration; however, the procedure must be performed quickly because they are cleared rapidly. Additionally, damage to the lymph vessels during surgery can allow the tracers to leak out, making SLN identification difficult. Furthermore, LNs in the deep pelvis are difficult to identify. Thus, tracers that are retained in the LNs, such as radioisotopes, play an important role. A meta-analysis showed that the SLN-detection rate is increased by a combination of radioisotope and dye^13,14^. However, radioisotopes are associated with exposure to radiation and patient compliance issues, making their management complicated.

It has been known that magnetic particles, such as superparamagnetic iron oxide (SPIO), are phagocytosed by macrophages in LNs and thus taken up into them and can then be detected by magnetic resonance imaging (MRI)^15,16^. LN metastasis may impair SPIO uptake due to lymph flow disturbance and destruction of the LN structure^17^. SPIO administration followed by MRI has been reported to be useful for the diagnosis of LN metastases in lung^18^, head and neck^19^, prostate and bladder^17,20^, rectal^21,22^, uterine^23^, and breast cancers^16^. All these previous reports, except for the one on breast cancer, involved diagnosis of LN metastasis by systemic SPIO administration.

SPIO can act as a retention-type tracer for SLN mapping because it remains within the LNs^24–26^. Compared with radioisotopes, SPIO is easy to handle and has few adverse effects. Local injection of SPIO as a tracer has been reported in patients with breast cancer^24^, and its usefulness has been demonstrated in clinical trials^25–32^. However, although the effectiveness of a magnetic detection system has already been reported for breast cancer^33^, there is no information on the use of SPIO in patients with uterine cancer.

The current pilot study aimed to examine the usefulness of SPIO injection into the uterine cervix, together with MRI and iron staining, for detecting SLNs and diagnosing LN metastasis in patients with uterine cancer.

## Results

### Identification of LNs by SPIO and radioisotope

**Figure 1:**
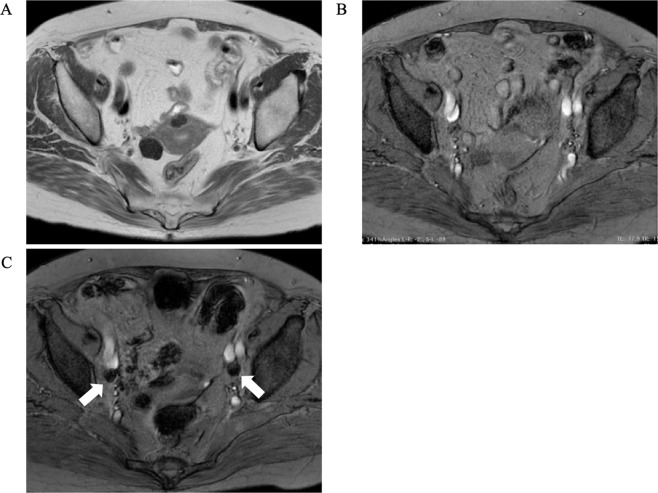
Representative SPIO/MRI images. (**A**) Pre-SPIO MRI T2-weighted image (axial); (**B**) pre-SPIO MRI T2*-weighted image (axial); (**C**) post-SPIO MRI T2*-weighted image (axial). LNs that have taken up SPIO show as enhanced black (white arrows). LN: lymph node; MRI: magnetic resonance imaging; SPIO: superparamagnetic iron oxide.

**Figure 2:**
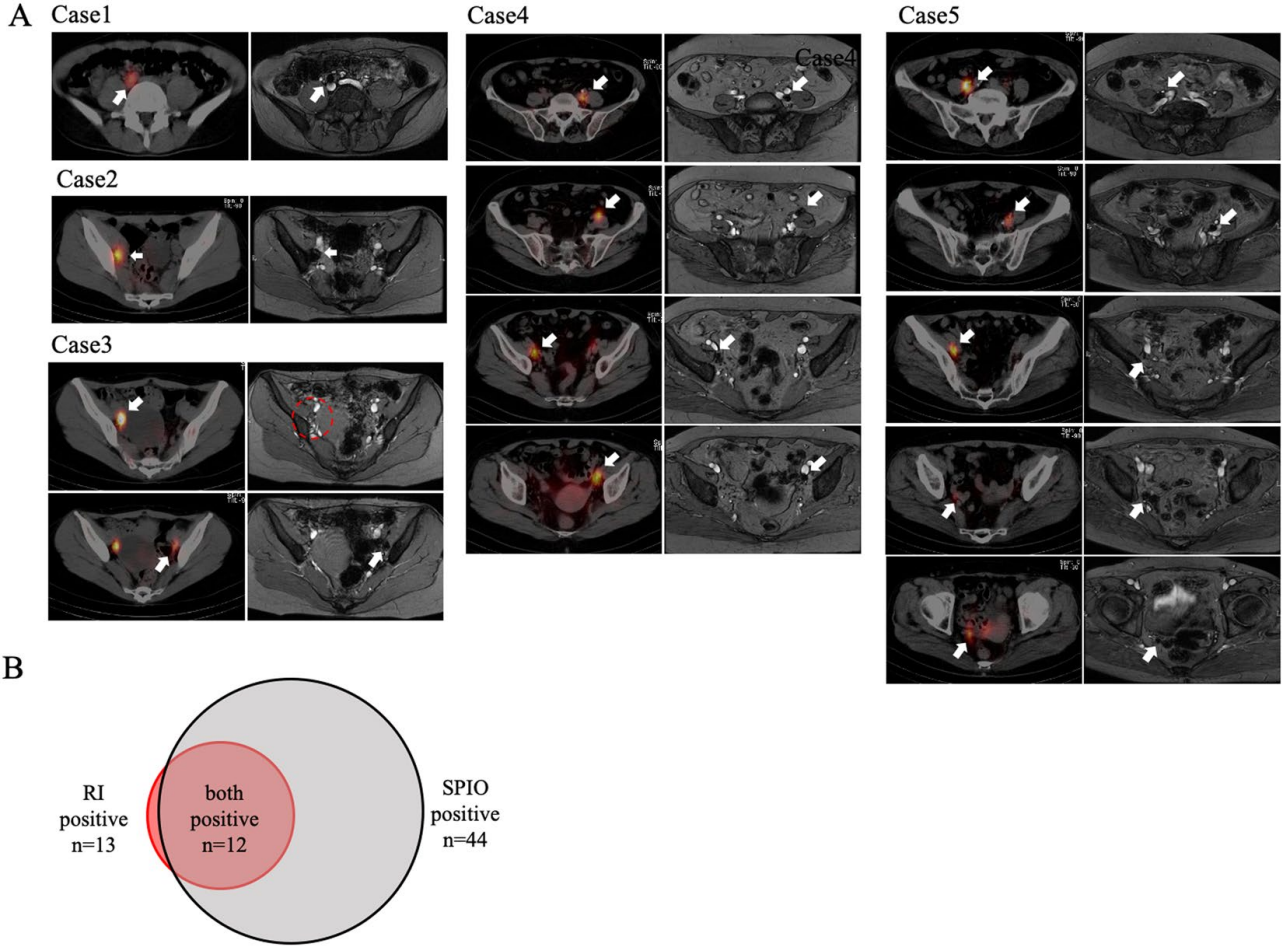
Images from five patients who underwent SPECT/CT and SPIO/MRI. (**A**) Comparison between SPECT/CT (left) and SPIO/MRI (right). Only the radioisotope-positive LN in Case 3 was not enhanced by SPIO/MRI (red dotted circle). (**B**) Relationship between radioisotope-positive and SPIO/MRI-positive LNs. Twelve of the 13 radioisotope-positive LNs were also positive for SPIO/MRI. LN: lymph node; MRI: magnetic resonance imaging; SPECT/CT: single-photon emission computed tomography/computed tomography; SPIO: superparamagnetic iron oxide.

SPIO/MRI was performed 2 days before surgery and radioisotope injection and SPECT/CT 1 day before surgery in five patients with cervical or endometrial cancer scheduled for surgical procedures that included pelvic LN dissection (Fig. 1). Details of these five cases are shown in Table 1. Forty-four LNs were SPIO/MRI-positive and 13 were radioisotope-positive. Of the 13 radioisotope-positive LNs, 12 (92%) were also SPIO/MRI-positive (Fig. 2A, B).

**Table 1:** Concordance of lymph node detection between SPECT/CT and SPIO/MRI.

Case	Cancer	FIGO stage	RI-positive LN		SPIO-positive LN	
			R	L	R	L
1	C	IB1	1	0	3	6
2	C	IB1	1	0	3	6
3	C	IB1	1	1	0	1
4	E	IA	1	3	7	8
5	E	IA	4	1	5	5
		total	8	5	18	26

### SPIO/MRI and iron staining

**Figure 3:**
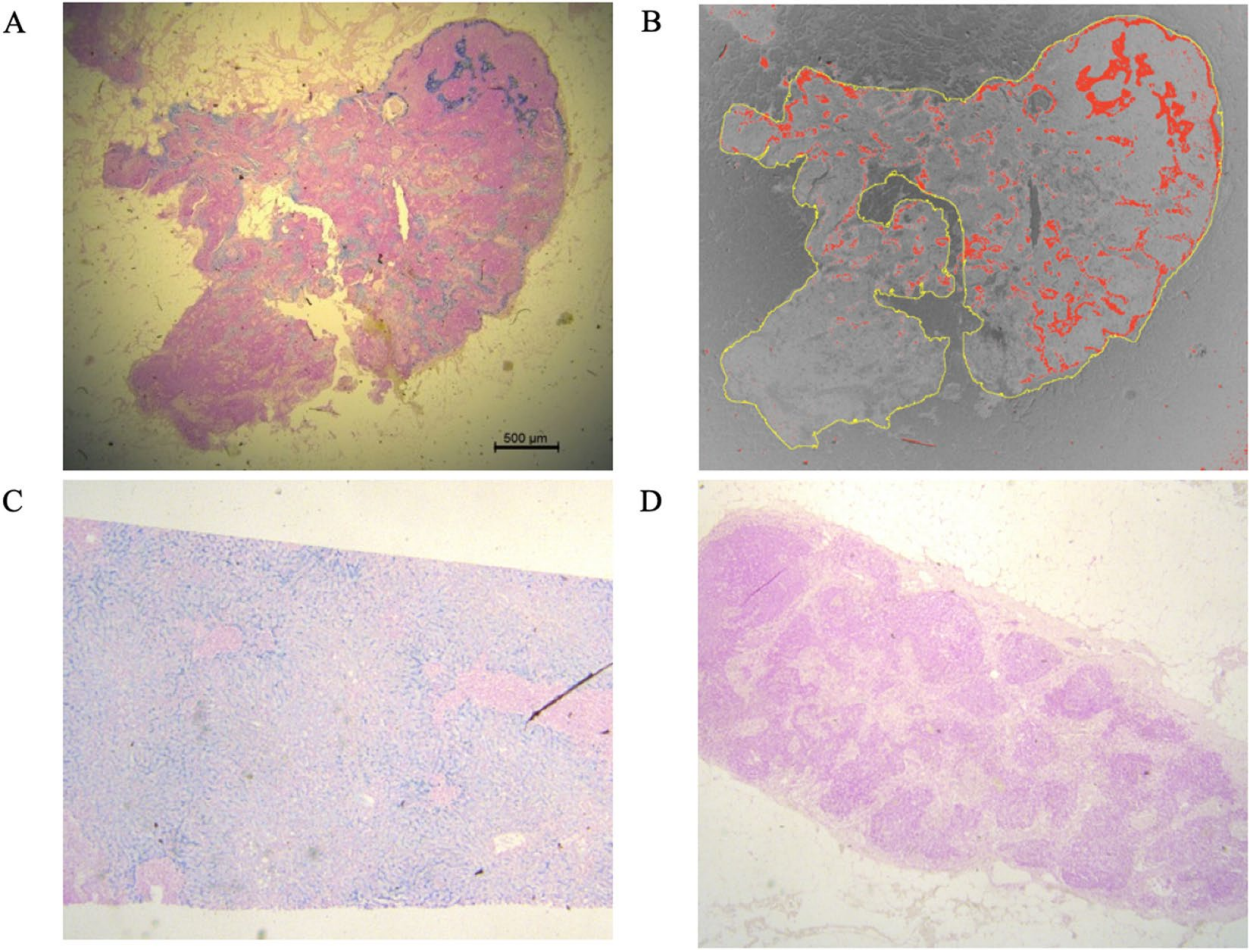
Example of iron-stained images and image analysis. (**A**) Iron stain-positive LN (×12.5). The part with SPIO uptake has stained blue. (**B**) Image analysis by ImageJ. The ratio of blue-stained area to the entire LN was defined as the staining ratio (%). Outline of LN indicated in yellow, iron stain-positive area displayed in red. (**C**) Positive control (liver) (×12.5); (D) Iron stain-negative LN (×12.5). LN: lymph node.

Dissected LNs from 15 patients who had undergone SPIO/MRI were subjected to iron staining (Fig. 3). Details of these patients are shown in Table 2. No adverse effects of SPIO injection were identified.

**Table 2:** Characteristics of 15 patients with uterine cancer.

	n	%
Age, years (median)	59 (31–76)	
**Cervical cancer**		
IB1	3	20
**Endometrial cancer**		
IA	6	40
IB	2	13
IIIA	1	7
IIIC2	2	13
IVB	1	7
**Days from SPIO injection to operation**		
1	3	20
2	9	60
3	2	13
6	1	7
**median: 2**		
	**median**	**range**
Number of isolated LNs in each case	38	20–49
Number of SPIO/MRI-positive LNs in each case	7	0–15
Number of iron-stain-positive LNs in each case	8	0–22

**Figure 4:**
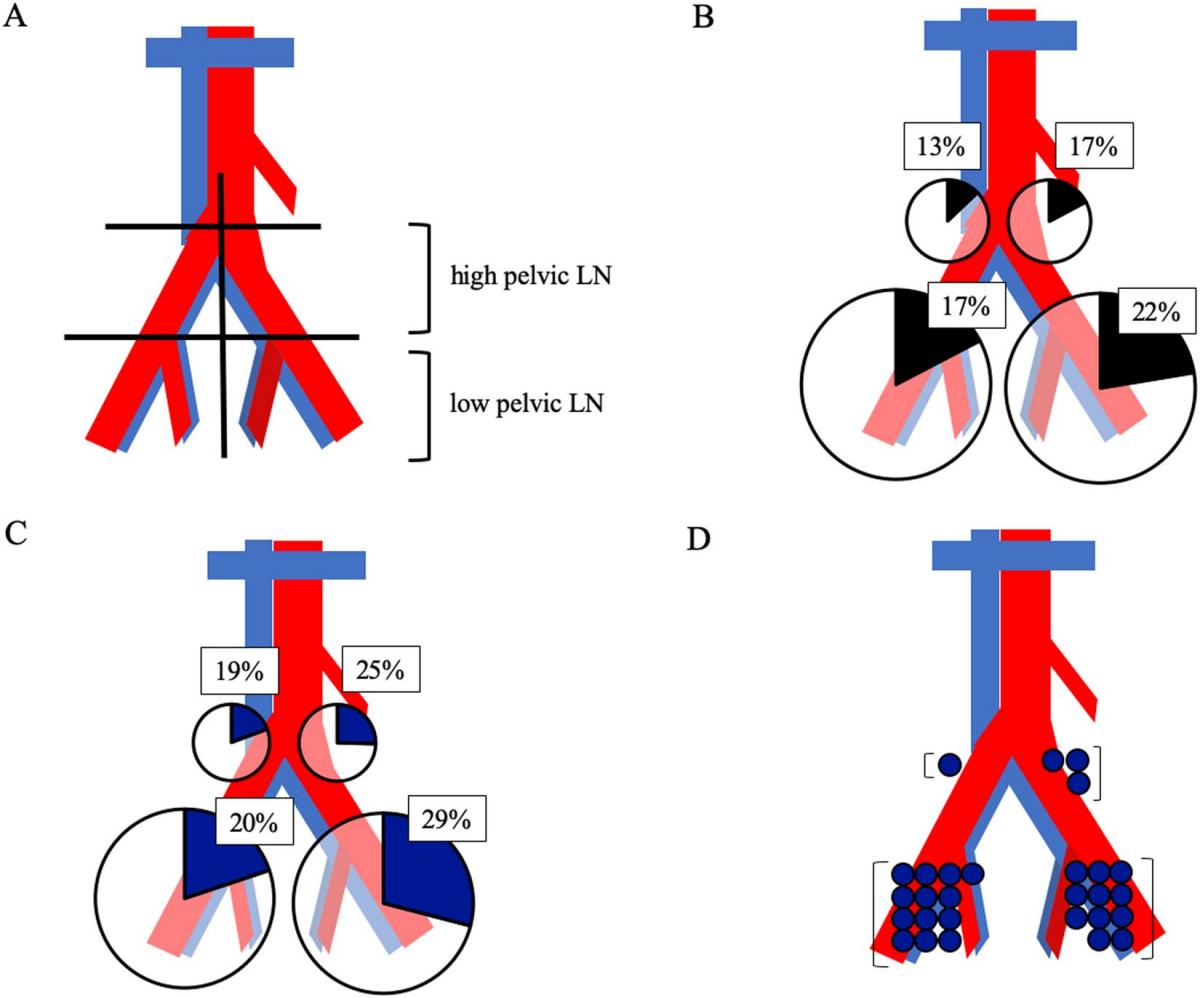
Distribution of SPIO/MRI-positive and iron-stain-positive LNs. (**A**) The pelvis was divided into left and right sides, and into upper (aortic bifurcation to common iliac bifurcation) and lower pelvic LN areas (distal to common iliac bifurcation). (**B**) Percentage of SPIO/MRI-positive LNs in each area; (**C**) Percentage of iron-stain-positive LNs in each area. (**D**) LN areas with the highest staining ratios are indicated by blue circles (30 pelvic sides in 15 cases). LNs in the upper area showed the highest staining in four of the 30 sides of the pelvis (13%). There were no iron-stain-positive LNs on either side in one case. LN: lymph node; MRI: magnetic resonance imaging; SPIO: superparamagnetic iron oxide.

The pelvis was divided into left and right sides, and further into upper (aortic bifurcation to common iliac bifurcation) and lower pelvic LN areas (distal from common iliac bifurcation) (Fig. 4A). The ratios of SPIO/MRI-positive LNs were similar among these four areas (χ^2^, p = 0.31) (Fig. 4B). The ratios of iron stain-positive LNs were also similar among the four areas (χ^2^, p = 0.14) (Fig. 4C). Furthermore, LNs with the highest staining ratios were located in the upper pelvic area in 3/15 (20%) patients and 4/30 (13%) sides (Fig. 4D).

**Figure 5:**
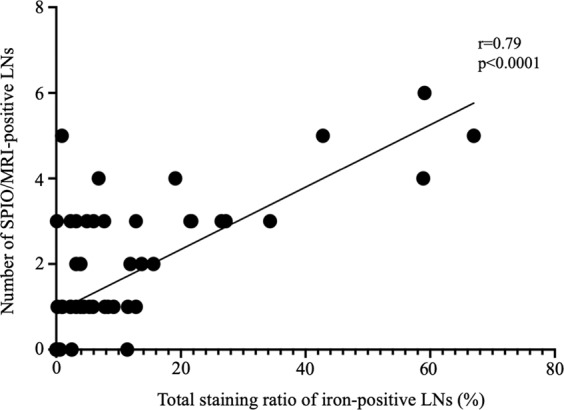
Correlation between SPIO/MRI and iron staining. The pelvises of 15 cases were divided into 60 areas (left, right, upper, and lower) and the total number of SPIO/MRI-positive LNs in each area and ratio (%) of iron-stain-positive LNs were calculated. There was a strong positive correlation between the number of SPIO/MRI-positive LNs and total staining ratio (Spearman’s rank correlation analysis, r = 0.79, p < 0.0001). LN: lymph node; MRI: magnetic resonance imaging; SPIO: superparamagnetic iron oxide.

Sixty areas from 15 patients (left or right side, and upper or lower area) showed highly positive correlations between the number of SPIO/MRI-positive LNs and the total staining ratio in each area (Spearman’s rank correlation coefficient, r = 0.79, p < 0.0001) (Fig. 5).

### Relationship between iron staining and sentinel LNs

**Figure 6:**
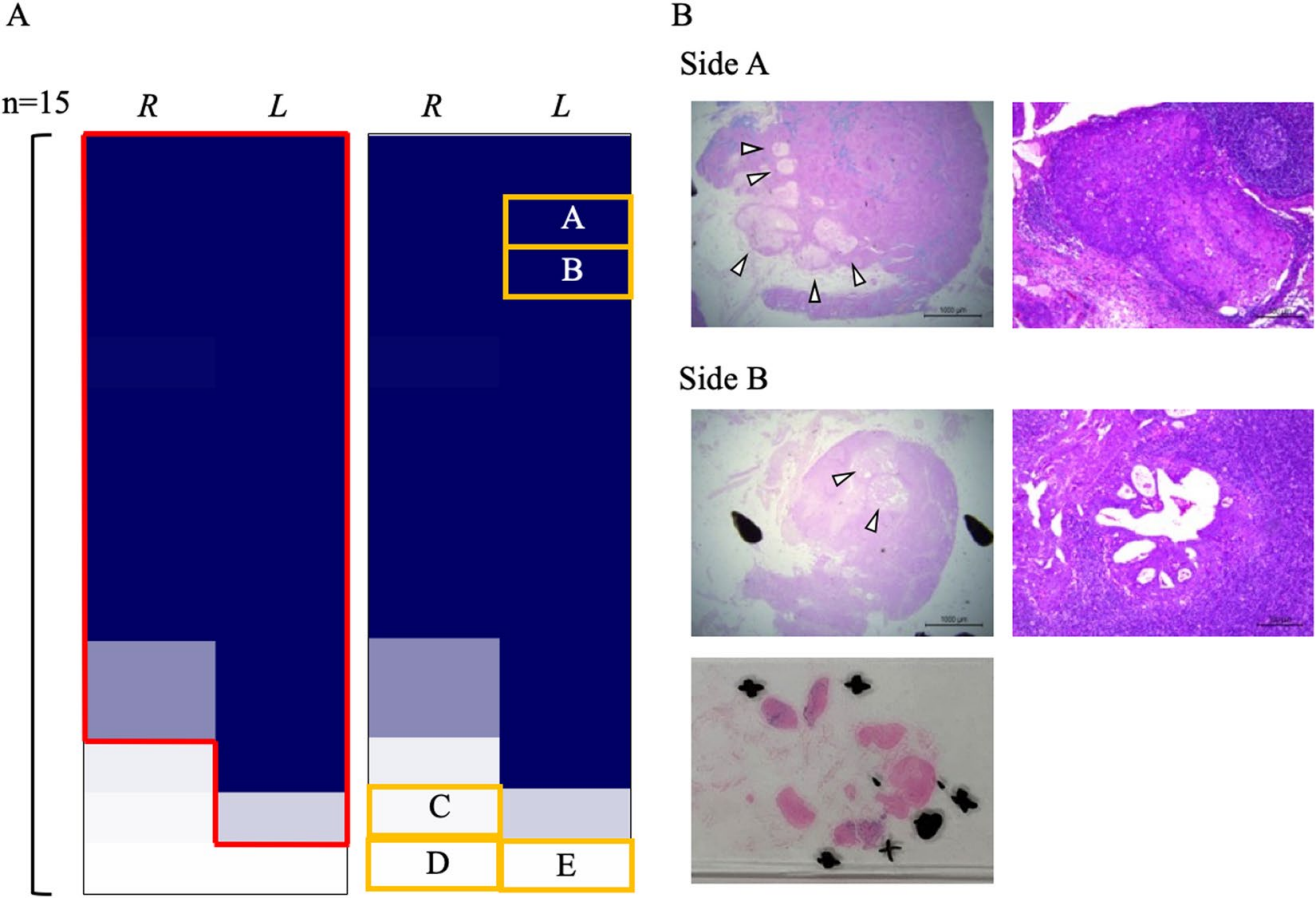
Relationship between iron-staining and sentinel LNs. (**A**) Total staining ratio on each side (left or right) of pelvis in 15 cases shown in a heat map. Red frame indicates sides with a total staining ratio ≥0.9%; yellow frames indicate sides with LN metastasis. (**B**) Photomicrographs. Side A: iron staining of LNs harbouring metastases (left panel, ×12.5, arrowheads indicate metastases) and HE staining (right panel, ×100), indicating metastasis of cervical cancer (squamous cell carcinoma). Iron staining is strong except for in the metastasis, suggesting that it is an SLN. Side B: iron staining of LNs harbouring metastases (left upper panel, ×12.5, arrowheads indicate metastases), HE staining (right panel, ×100), and image of the preparation including surrounding LNs (black circles show LNs harbouring metastases, + shows iron-stain-positive LNs), indicating metastasis of endometrial cancer (endometrioid carcinoma G1). The LN harbouring metastases shows weakly positive iron staining, whereas the nearby LNs are strongly stained, suggesting that this area contains the SLN. HE: haematoxylin and eosin; LN: lymph node.

The staining ratio was totalled and the findings shown as a heat map for 30 sides (left or right) of pelvis from 15 patients (Fig. 6A). The total staining ratio was ≥0.3% in 26/30 (87%) sides of the pelvis. Additionally, the SLN on at least one side was identified by iron staining in 14/15 (93%) cases.

There were LN metastases in five sides of the pelvis in four patients. Details of two (Sides A and B) are shown in Fig. 6B. There was a LN metastasis in Side A; the remainder of the LN was well-stained (staining ratio: 6.7%). Iron staining was strong except for within the metastasis, suggesting that it was an SLN. There was also a LN metastasis in Side B. Five iron stain-positive LNs were identified in paraffin sections, four of which were strongly positive (staining ratios: 19.0%, 12.8%, 12.1%, 8.3%), the other being weakly positive (staining ratio: 1.5%) and harbouring a metastasis. The LN with a metastasis showed weakly positive iron staining, whereas the nearby LNs were strongly stained, suggesting that this area contained the SLN. There were no iron stain-positive LNs in the other three sides of pelvis, or only one very weakly positive LN (staining ratio: 0.1%), and the LN harbouring a metastasis was negative for iron staining in these sides of pelvis. We concluded that LN metastasis may have impaired the lymph flow and disrupted the LN structure on affected sides of the pelvis.

## Discussion

In this study, we injected radioisotope and SPIO into the cervixes of patients with uterine cancer and compared the results of SPECT/CT and SPIO/MRI. SPIO and the radioisotope were taken up similarly by LNs; however, SPIO was taken up by more LNs than the radioisotope, suggesting that using SPIO alone may achieve detection of many SLNs. The use of SPIO in combination with a dye or ICG may thus increase the detection sensitivity for SLNs. Further studies are needed to determine whether radioisotopes or SPIO are superior in terms of sensitivity and specificity.

In this study, we carried out iron staining of formalin-fixed, paraffin-embedded LNs to determine the uptake of SPIO qualitatively and quantitatively. We showed that SLNs could be identified pathologically, thus providing the first report of a pathological diagnosis by iron staining of LNs after administration of SPIO. It is often difficult to detect SLNs accurately and do rapid pathological examinations during surgery^35,36^; thus, postoperative evaluation of SLNs in permanent specimens may aid in making accurate pathological diagnoses. To the best of our knowledge, there are no reported objective and quantitative studies of retention time of SPIO in LNs after local injection. In the current study, SPIO was not washed out of the LNs. Even when the local injections had been administered several days before surgery, SPIO could still be detected by iron staining. Because our study cohort was small, further studies are needed; however, we can conclude that the schedule for administering SPIO could be relatively flexible in clinical practice.

There was a strong positive correlation between the number of SPIO/MRI-positive LNs and the total staining ratio, indicating that iron staining reflects preoperative SPIO uptake and that SLNs can be predicted by SPIO/MRI. Interestingly, SPIO/MRI and iron staining showed that SPIO was taken up by the upper (common iliac and sacral LNs) and lower pelvic LNs (internal and external iliac and obturator LNs) at comparable rates. However, in some cases SPIO was taken up more strongly by the upper than the lower pelvic LNs. In patients with uterine cancer, the SLNs are often the obturator and external iliac LNs^37,38^. However, lymph flow has sometimes been reported to run cephalad along the mesoureter and SLNs may thus include the common iliac LNs^39,40^. Our results indicate that upper pelvic LNs can become SLNs via this lymph flow, suggesting that it is important to search for both upper and lower pelvic LNs. Our results also suggest that more SLNs may have been detected in the lower pelvic LN because there are more LNs in this area; however, the rate of SLNs was the same in the upper and lower pelvic LNs.

We found metastases in LNs in five out of 30 sides (left or right) of the pelvis examined in 15 patients, including two sides with high total staining ratios and SLN metastasis. SLNs are more likely to occur in areas with SPIO/MRI- or iron stain-positive LNs. Pathological examination is thus recommended in the event of strongly positive iron staining. LN metastasis is known to disturb the lymph flow, sometimes preventing detection of SLNs^14^. In the current study, the three sides of the pelvis with negative or weak iron staining contained LN metastases; however, the SPIO and lymph flows may have been impaired by the tumour. Adequate LN dissection may thus be needed on any side of the pelvis in which no SPIO/MRI-positive LNs are detected and a pathological examination may be required on any side of pelvis with poor iron staining. This would confirm adequate LN dissection by checking SPIO/MRI-positive LNs and comparing the results of iron staining.

Three sides of the pelvis in two patients contained pathologically-confirmed LN metastases, including one patient with LN metastases on both sides of the pelvis who demonstrated SPIO/MRI- and iron stain-negative LNs and in whom SPECT/CT failed to detect any LN metastases. SPIO/MRI may thus be useful for diagnosing LN metastasis. However, in the patient with LN metastasis on only one side of the pelvis, the LN metastasis was in the upper area, and only one very weakly positive iron staining (staining ratio: 0.1%) LN was detected in the lower area. This side contained an SPIO/MRI-positive LN, possibly reflecting detection of very little iron uptake by MRI. Moreover, because LNs with micrometastases were positive for iron staining, it was not always possible to diagnose LN metastasis on the basis of contrast defects in SPIO/MRI, as in previous reports^16^.

Regarding limitations, this was a pilot study with a small number of cases; thus, further studies with larger numbers are needed to confirm the results. It is also necessary to determine how many days SPIO stays in SLNs. In addition, because patients in whom SLN biopsy is indicated often undergo endoscopic surgery, it is necessary to develop magnetic detectors that are suitable for use in this type of surgery. It is also necessary to examine further the possibility of identifying and removing the SLN alone to reduce overall LN dissection.

In conclusion, SPIO and radioisotopes are taken up similarly by SLNs in patients with uterine cancer. SPIO/MRI and iron staining may be useful techniques for identifying SLNs and diagnosing LN metastasis in patients with uterine cancer.

## Materials and Methods

### Patients

We enrolled patients with cervical or endometrial cancer who were scheduled for surgery, including pelvic LN dissection up to the aortic bifurcation, at Kindai University Hospital from February 2016 to March 2019, and who provided informed consent. Patients were excluded if they were under 20 years of age, did not provide consent, or had received preoperative chemotherapy. The expected sensitivity for identification of SLNs was assumed to be 80% and the threshold to be 50%. The sample size was calculated by setting the significance level to one side 0.05 and the power to 0.8; the required number of cases was 18. This study was approved by the Institutional Review Board of Kindai University Faculty of Medicine (26-206, 27-095) and registered in the UMIN Clinical Trials Registry (UMIN000023607, date of registration; 12/08/2016). All research was performed in accordance with Ethical Guidelines for Medical and Health Research Involving Human Subjects.

### SPIO injection

Ferucarbotran (Resovist; Fujifilm Toyama Chemical Corporation, Tokyo, Japan) is intravenously administered at 0.45 mg/kg as iron when used as a contrast agent for MRI. In a preliminary study, when 10 mL of saline was added to 1.6 mL of ferucarbotran (total 11.6 mL, including 44.6 mg as iron) to produce almost the same amount as that used for intravenous administration and 6 mL (total 24.0 mg as iron) was injected into the uterine cervix, the cervix was too strongly enhanced and there was too much artefact (data not shown). Then, when ferucarbotran was administered in the same volume but in about half the concentration, the MRI images were fine with little artefact around the cervix and satisfactory imaging of LNs. Therefore, this amount was set as the dose in this study. Hence, 1.6 mL (including 44.6 mg as iron) was mixed with 20 mL saline to a total volume of 21.6 mL and administered several (1–6) days before surgery. Details of the days of administration are shown in Table 2. Cattelan 23 G needles were inserted into the subepithelium of the cervix (1, 5, 7, 11 o’clock), and 1.5 mL was injected smoothly through each needle.

### SPIO/MRI protocol

Pre-SPIO images were constructed as described below. MRI was repeated 3 h after SPIO injection, after which post-SPIO images were constructed using the same protocol.

Images were obtained using a 1.5 T system (Signa HDxt; GE Healthcare Japan, Tokyo, Japan) with an HD 12-channel body array coil. T2-weighted axial images were obtained with the following parameters: repetition time, 3000 ms; time of echo, 75 ms; echo train length, 30; NEX, 2; matrix, 416 × 416; field of view, 270 mm; slice thickness, 5 mm; and number of acquisitions, 30. T2*-weighted axial images were obtained as follows: repetition time, 150 ms; time of echo, 17.9 ms; flip angle, 50°; matrix, 320×256; field of view, 280 mm; slice thickness, 5 mm; and number of acquisitions, 48. T2*-weighted sagittal images were obtained using the following parameters: repetition time, 150 ms; time of echo, 17.9 ms; flip angle, 30°; matrix, 320×250; field of view, 270 mm; slice thickness, 4 mm; and number of acquisitions, 34.

### Radioisotope injection

^99m^Tc (Techne Phytate Kit; Fujifilm Toyama Chemical Corporation) was injected on the day before surgery using the above-described technique with 0.2 mL (15 MBq) per needle (total 60 MBq ^99m^Tc), with careful attention to exposure.

### Single-photon emission computed tomography/computed tomography (SPECT/CT) protocol

SPECT/CT was performed every 30 min after radioisotope injection until hot LNs were detected on either side of the pelvis, or for a maximum of 2 h. The SPECT/CT system (Symbia T6; Siemens, Erlangen, Germany) consists of a dual-head, variable-angle gamma camera equipped with low–medium energy general purpose collimators and a six-slice spiral CT scanner optimized for rapid rotation. SPECT acquisition (matrix, 128 × 128; 60 frames at 7 s/view) was performed using steps of 6°. CT scanning was performed in low-dose mode for attenuation correction and 5-mm slices were created. Iterative reconstruction (3D-ordered subset expectation maximization) was used to generate SPECT slices. SPECT images and CT axial slices were fused using an e-soft application package (Siemens) and hybrid SPECT/CT images were viewed using two-dimensional orthogonal re-slicing in axial orientation.

### Iron staining

Paraffin-embedded sections (3 μm) of dissected LNs were deparaffinized, rinsed, and soaked in Berlin Blue (25 mL 2% potassium hexacyanoferrate [II] trihydrate; Fujifilm Wako Pure Chemical Corporation, Osaka, Japan) and 25 mL hydrochloric acid (Sigma-Aldrich, Tokyo, Japan) for 30 min. Specimens were rinsed again, and soaked in 0.1 g Nuclear Fast Red (Sigma-Aldrich) dissolved in 100 mL heated aluminium sulphate 14-18 water (Fujifilm Wako Pure Chemical Corporation) for 2 min, and then rinsed, dehydrated, permeated, and sealed.

### Staining ratio

LN images were captured at ×12.5 or ×25 magnification according to the size of the LN using a microscope camera (Leica DMC2900; Leica Microsystems, Tokyo, Japan) and software (Leica Application Suite ver. 4.5; Leica Microsystems). Images were captured separately for large LNs so that the ranges did not overlap. The captured images were saved in JPEG format, imported to ImageJ^34^, and split into red, green, and blue. After binarizing the green images and adjusting the threshold, the area of the entire LN was measured. Images were then created by subtracting red from blue and the threshold adjusted so that the iron-stained blue area to be extracted became red. The area was measured and the ratio (%) of the iron-stained area to the total area of the LN determined and defined as the staining ratio.

### Statistical analysis

Statistical analysis was carried out using GraphPad Prism ver. 8.2.0 (GraphPad Software, San Diego, CA, USA). The χ^2^ test and Spearman’s rank correlation analysis were used for statistical analysis. A probability (P) value of <0.05 was considered to denote statistical significance.
